# The relationship between problematic Instagram use and eating disorders psychopathology: an explanatory structural equation model

**DOI:** 10.1007/s00127-023-02477-1

**Published:** 2023-04-06

**Authors:** Giulia Fioravanti, Emanuele Cassioli, Eleonora Rossi, Lorenzo Lucherini Angeletti, Silvia Casale, Valdo Ricca, Giovanni Castellini

**Affiliations:** 1 Psychology Unit, Department of Health Sciences, University of Florence, Florence, Italy; 2 Psychiatry Unit, Department of Health Sciences, University of Florence, Largo Brambilla, 3, 50134 Florence, Italy

**Keywords:** Body uneasiness, Eating disorders, Physical appearance, Problematic Instagram use, Social comparison, Structural equation model

## Abstract

An emerging body of research has evidenced the negative influence of using and being exposed to social networking sites (SNSs) on body image. Furthermore, it has been postulated that SNS use might be related with onset and persistence of eating disorders (EDs) psychopathology. The aim of the present study is to evaluate the complex interplay between problematic Instagram use (PIU) (conceptualized as a potential behavioral addiction comprising withdrawal, conflict, tolerance, salience, mood modification and relapse) and ED psychopathology, by means of an explanatory structural equation model. We hypothesized that PIU would be associated with ED symptoms through the mediating role of appearance comparison, individual psychological investment in physical appearance, and body uneasiness. A sample of 386 young female participants (M_age_ = 26.04 ± 6.73) was recruited, of which 152 had received a diagnosis of ED. ED patients used Instagram more than the control group and showed higher levels of PIU. Results from structural equation modeling (fit indices: χ^2^ = 44.54, df = 19, p < 0.001; RMSEA = 0.059; CFI = 0.98; SRMR = 0.02) showed that PIU predicted appearance comparison and psychological investment in physical appearance, which in turn predicted body uneasiness. In turn, body uneasiness predicted ED psychopathology and interpersonal difficulties. Our model provides a useful account of how eating disorder symptoms could be triggered and maintained by an addictive use of Instagram.

## Introduction

Eating disorders (EDs) are impairing and chronic psychological disorders characterized by predisposing and maintaining biopsychosocial factors, such as low body weight, disturbances in body image and eating behaviors, and interpersonal difficulties [1, 2]. A central aspect of ED psychopathology revolves around bodily-related concerns, such as body dissatisfaction and body uneasiness [, 1, 2]. The latter involves a cognitive-affective disposition toward one's body and represents an inclusive assessment of bodily-related concerns, focusing not only on aspects of satisfaction/dissatisfaction with specific body parts, but also consequent behaviors such as avoidance, compulsive checking behaviors (e.g., self-monitoring in the mirror), and related emotions such as anxiety, distrust, and detachment which are concurrent in engendering a feeling of alienation from one’s own body [3]. Accordingly, body uneasiness seems to be more resistant to treatment [4, 5], besides being associated with a worse prognosis [2, 6, 7] and a higher likelihood of relapse after remission [, 8, 9].

Bodily-related concerns can be influenced by sociocultural factors, with mass media being the most impactful one [10]. Ideal body shapes conveyed by traditional mass media, such as magazines and television, comprise unattainably thin and toned bodies, exalting slenderness, and weight loss [11]. The relationship between traditional media exposure and bodily-related concerns has been supported by a large number of correlational and experimental studies both among women [12] and men [13]. According to the sociocultural theory of body dissatisfaction [14] there are two mechanisms involved in this relationship: (i) internalization of appearance ideals (e.g., thin, muscular, and fit ideals); (ii) appearance-based social comparison. Specifically, frequent media exposure leads individuals to internalize the “ideal thin” as beautiful and desirable, and compare themselves to these idealized images, engendering a dissatisfaction towards their body and appearance [15]. Nevertheless, albeit body uneasiness involves a comprehensive behavioral and emotional spectrum, its specific relationship with frequent media exposure is still unclear.

Although traditional media are still largely used, other types of “new” media are being increasingly diffused, most evidently the social networking sites (SNSs). SNSs are online platforms where users can create and share content with other users [16]. These sites differ from traditional media in two main aspects: (i) they are interactive; (ii) the content is mostly generated by peers [17]. Users are simultaneously information sources and receivers, they can actively decide their participation by creating their own profiles and posts, browsing the information posted by other users and interacting with them by means of “likes” and comments. The negative influence of using and being exposed to SNSs on body image has been recently evidenced by an emerging body of research. Specifically, a wide number of studies have found that SNS use is associated with body dissatisfaction and disorderly eating among young women and men (for a systematic review, see [18, 19]; for a meta-analysis, see [20]). Internalization of beauty ideals, appearance-based comparison and self-objectification were found to explain the detrimental effect of SNS use on body image [18], providing support for both the social comparison [14] and objectification theory (i.e., internalization of an observer's perspective as the primary view of the physical self) [21] in the field of SNSs. Indeed, like traditional media, SNSs are often appearance-focused since users post photos in which they look good and attractive, enhanced by the application of filters or digital editing tools, and composed [22]. As a result, many of the presented images on SNSs are idealized and unrealistically attractive, which may have a role in inducing body dissatisfaction. Moreover, although the majority of the studies have investigated the effect of the exposure to idealized images on SNSs on the general/evaluative component of body image (i.e., body satisfaction/dissatisfaction), those studies that have investigated the impact of SNSs on cognitive (appearance self-esteem and psychological investment in physical appearance) and behavioral (disordered behaviors related to body image) dimensions of body image found higher effect sizes [20].

Noteworthy, studies that assessed the relationship between appearance-focused social media use and body image obtained a stronger effect size than studies that investigated general social media use [18, 20], suggesting that future research would benefit from investigating more image-based SNSs, such as Instagram. In addition to its raising popularity, compared to other popular SNS such as Facebook or Twitter, Instagram uniquely focuses on image-based content such as photo and video updates, and provides an in-built tool that offers several possible filters to enhance the appearance of a photo. Thus, Instagram users are not only more likely to promote an idealized self-image but also to be exposed to other users’ idealized images (rather than real ones), and this could increase upward appearance-based comparisons and their negative effects on body image. An increase in body dissatisfaction following exposure to idealized Instagram images of thin and attractive women relative to control images was found [23–25]. Moreover, the exposure to fitspiration images (i.e., people usually exercising, or dressed in exercise outfits) was also associated with the development of body image dissatisfaction [26–29]. Indeed, content analyses showed that fitspiration images, albeit focused on fitness and health, foster weight loss and place particular value on physical appearance, depicting only thin and toned models [30]. A recent systematic review examining the state-of-the art on the relationship between Instagram use and different mental health indicators, obtained most evidence for the relationship between intensity of Instagram use and social comparison, body dissatisfaction, dietary behaviors, and disordered eating outcomes [31]. Finally, Griffiths et al. [32] found that exposure to both thinspiration and fitspiration contents on image-centric social media was associated with more frequent physical appearance comparisons, and through these, greater symptom severity among a clinical sample of individuals with EDs. These results, as a whole, suggest that the likelihood of developing EDs symptoms might be enhanced among those people reporting a lack of self-regulation in one’s own use of Instagram, often named problematic Instagram use (PIU). Based on the six components model of Griffiths [33], PIU has been defined as a maladaptive engagement with SNSs leading to impairment in personal, social, and vocational/academic functioning. More precisely, although it is not formally acknowledged or embedded in current psychiatric nosology [1], PIU represents an excessive and compulsive online social networking behavior, which shares common symptoms of well-recognized chemical and behavioral addictions (e.g., substance addiction, sex addiction): salience, tolerance, mood modification, conflict, withdrawal, problems, and relapse [34, 35]. Hence, one may presume that an addictive use of Instagram might have major impacts on social, emotional, and cognitive domains. Very recent studies have shown that PIU negatively impact body esteem through the mediating role of physical appearance perfectionism, that is, concerns about imperfection [36], in keeping with previous findings supporting a positive association between problematic social networking sites use and lower body and self-esteem and higher ED symptoms/concerns [37]. Moreover, PIU was found to be associated with elevated body image dissatisfaction which in turn was related to increased psychopathological symptoms [38]. Interestingly, a recent meta-analysis postulated that Internet addiction may have gender-specific distinctions. Thus, while men were more likely to exhibit internet gaming disorder, women reported a greater vulnerability to display social media addiction [39]. PIU would then appear to be a prevalent addictive behavior among women, consistently with a higher incidence rate of eating disorders in the female gender itself.

To date we are unaware of any study that has specifically examined the association between PIU and EDs psychopathology. Research on this topic is relevant because there are multiple reasons to believe that a compulsive and unregulated use of Instagram might be related with onset and persistence of EDs psychopathology. In particular, body uneasiness as a central aspect of ED psychopathology, as aforementioned, can be induced by exposure to idealized images found on SNSs, especially on those focused-on appearance-based content, such as Instagram. Despite the lack of empirical evidence concerning the relationship between PIU and body uneasiness, the frequency of Instagram use has been reported to predict higher body dissatisfaction [40, 41]. Therefore, it is reasonable to assume that body uneasiness might explain the relationship between PIU and eating disorders psychopathological symptoms. Indeed, a mediating role of body dissatisfaction in the relationship between PIU and different psychopathological outcomes including loneliness, depression, anxiety, and social anxiety was evidenced by a previous recent study [38]. Additionally, previous systematic reviews [18, 19] indicated appearance-based social comparison as one of the main explaining mechanisms of the negative effect of SNSs use on body image. According to social comparison theory, individuals tend to compare their opinions and abilities with others’ opinions and abilities [42]. Young women exposed to thin ideal body images appear to automatically engage in social comparison when viewing SNSs idealized images, thus inducing body dissatisfaction. Therefore, it is plausible to suppose that the appearance-based comparison might explain the relationship between PIU and body uneasiness. In addition, it was found that the relationship between appearance-focused social media use and bodily-related concerns was stronger when considering the cognitive dimensions of body image such as the psychological investment in physical appearance [20]. The latter reflects the cognitive-behavioral investment in one's appearance as an expression of the importance one attributes to personal appearance, and it has been associated with a higher prevalence in the female gender which appears to decrease with age [43]. It is possible that the continuous exposure to idealized images found on Instagram may lead females to attach more importance to the way they look like and consequently to feel more concerned about their body. For this reason, psychological investment in physical appearance might be a further underlying mechanism for the relationship between PIU and body uneasiness.

Despite the extant literature, it has not been empirically shown how PIU affects ED psychopathology both in general and in clinical populations. In addition, the role of PIU in the emergence of the interpersonal difficulties is also still unclear. The heightened levels of social anxiety exhibited by ED patients underscore the importance of interpersonal difficulties as risk and maintaining factors in these disorders, as supported through the association between interpersonal difficulties and body dissatisfaction [44, 45]. Intriguingly, studies indicated that the onset of social anxiety tends to precede the development of EDs [45, 46], suggesting that this factor may also represent a generative mechanism of EDs that needs to be taken into account. Moreover, even less is known about the mediating role of appearance-based comparison tendency, psychological investment in physical appearance and body uneasiness.

**Fig. 1:**
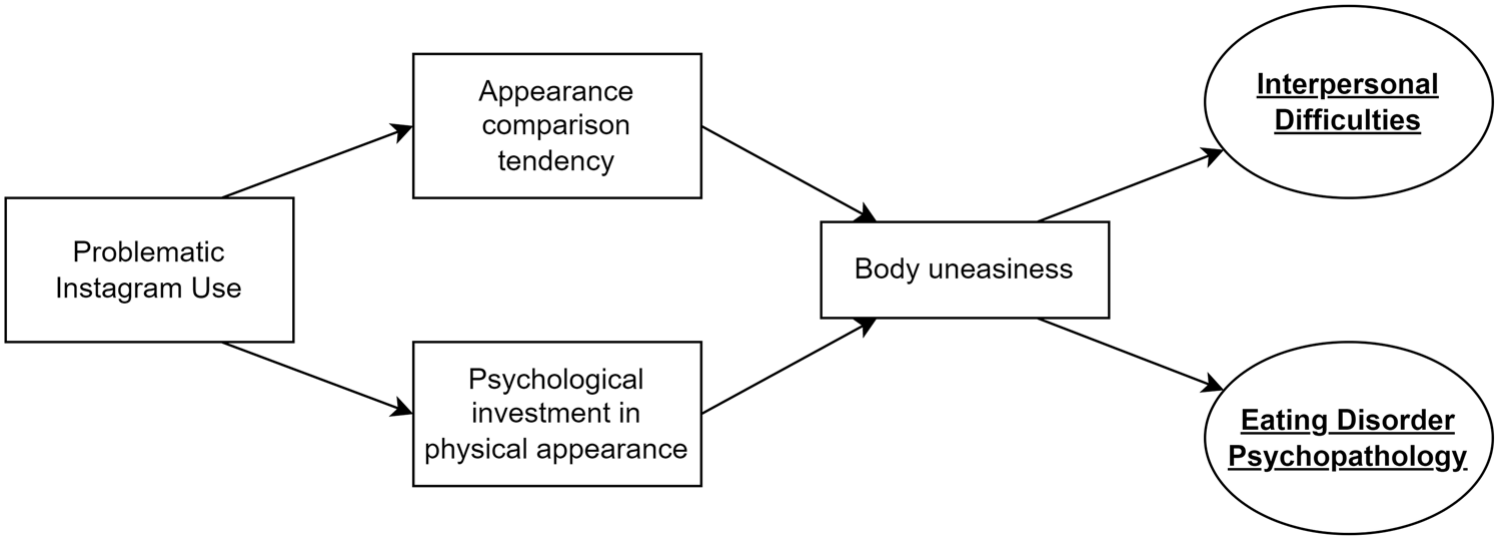
The hypothesized model

The current study aims to fill this gap by investigating PIU amongst individuals with eating disorders and comparing PIU levels in young women with and without EDs. Based on the above-mentioned evidence we expected women with ED symptoms to show more PIU levels, as well as a higher tendency to make appearance comparisons on Instagram, with respect to women without ED symptoms. Moreover, as no previous study has investigated the potential psychological processes underlying the link between PIU and EDs, the current study wanted to evaluate a model explaining how PIU relates to ED psychopathology on young women, considering the mediating role of specific dimensions of body image (i.e., psychological investment in physical appearance and body uneasiness) and appearance comparison tendency. More specifically, it was hypothesized that PIU would lead a person to make more frequent physical appearance comparisons and increase his/her psychological investment in physical appearance, thereby causing body uneasiness. Body uneasiness would, in turn, predict ED symptoms and related interpersonal difficulties (Fig. 1).

## Methods

### Participants

A sample of female patients suffering from EDs were recruited during their first outpatient evaluation at the Eating Disorders Clinic of Florence University Hospital, adhering to the following inclusion criteria: (i) female sex, (ii) age 18–65 years, (iii) current diagnosis of anorexia nervosa (AN), bulimia nervosa (BN) or binge-eating disorder (BED) according to the latest edition of the Diagnostic and Statistical Manual of Mental Disorder (DSM-5) [1], (iv) having an Instagram account and using it on a daily basis, (v) written informed consent for participation in the study and publication. Subjects were excluded from the study in the presence of illiteracy, intellectual disability, psychotic symptoms, or manic state at the time of enrolment. Control participants were enrolled through advertisement among attenders of the local University and online on popular social media (Facebook, Instagram, Twitter) using convenience snowball sampling. Inclusion criteria for controls were (i) female sex, (ii) age 18–65 years, (iii) having an Instagram account and using it on a daily basis, (iv) written informed consent for participation in the study and publication, whereas exclusion criteria were the presence of illiteracy or intellectual disability.

### Measures

Basic sociodemographic data were collected (sex, age, and education), along with information about the time spent on Instagram on a typical day. The following questionnaires were administered to all participants:

- Instagram Use Questionnaire (IUQ) [47]: this scale assesses PIU across nine items (e.g., “I feel anxious, when I am not able to check my Instagram account”) on a 5-point rating scale from “never” to “always,” covering withdrawal and compulsive use. Higher scores indicate higher levels of PIU. In its original validation study, this scale showed high internal validity (Cronbach’s α = 0.89) and very good fit indices in confirmatory factor analysis [47]. The IUQ was translated from English into Italian using a parallel back-translation procedure. A bilingual individual (D.B.) provided an Italian translation of the scale, and a second bilingual individual (A. S.) translated this version back into English. The forward- and back-translations were then assessed by a committee consisting of the same two translators and the first and the last author of this paper. None of the items raised any concerns. The Italian version of IUQ used in the present study demonstrated good reliability (Cronbach’s α = 0.87).
- State Appearance Comparison Scale (SACS) [48, 49]: A 3-item questionnaire for investigating appearance-related thoughts and comparisons with others engendered by exposure to Instagram images. Participants rate their agreement with each item on a 7-point rating scale (e.g., “To what extent did you think about your appearance while viewing Instagram images?”). Higher scores indicate higher levels of appearance comparison tendency. This scale has demonstrated good reliability [48]. The Italian version showed excellent internal consistency [49]. In the present study, the SACS demonstrated excellent internal consistency with a Cronbach’s α = 0.94.
- Appearance Schema Inventory-Revised (ASI-R) [50]: A 20-item self-report measure of individuals’ psychological investment in their physical appearance, including questions on grooming behaviors, social self-presentation and self-esteem. Participants respond on a 5-point rating scale from 1 = completely disagree to 5 = completely agree. Higher scores indicate higher degrees of psychological investment in one’s appearance. The Italian version [23] showed good reliability. Cronbach’s alpha in the current study was α = 0.89.
- Body Uneasiness Test A (BUT-A) [3]: A widely utilized 34-item questionnaire for the evaluation of body uneasiness over the following subdomains: weight phobia, body image concern, avoidance, compulsive self-monitoring and depersonalization. In addition to the abovementioned subscales, a total score (called Global Severity Index, GSI) can be computed. Each item is rated on a six-point rating scale (range 0–5, from “never” to “always”). Higher scores indicate greater body uneasiness. In the Italian validated adaptation, reliability coefficients of the scales ranged from 0.79 and 0.90 showing excellent internal consistency, good test–retest reliability (as the correlation coefficients were greater than 0.7), as well as a good predictive validity for AN (restrictive and binge-purging types) and for BN purging type [3]. This test showed excellent internal consistency in the present study (Cronbach’s α = 0.97).
- Eating Disorder Inventory 2 (EDI-2) [51, 52]: A self-report measure with 91 items and 11 subscales for the assessment of different ED-related domains: drive for thinness, bulimia, body dissatisfaction, ineffectiveness, perfectionism, interpersonal distrust, interoceptive awareness, maturity fears, asceticism, impulse regulation and social insecurity. Each item is rated on a six-point rating scale (from “never” to “always”). Higher scores indicate higher psychopathology. The Italian validated version demonstrated good internal consistency when administered to patients with an eating disorder: in this case, a Cronbach’s alpha coefficient ranging between 0.78 and 0.84 was obtained, whereas for non-patient subjects, internal consistency varied in a range between 0.38 and 0.88 (Cronbach’s alpha) [52]. Overall Cronbach’s α in the present sample was 0.93, indicating excellent reliability.

### Statistics

Patients were compared with control subjects for socio-demographic characteristics and study variables, using age-adjusted Analysis of Covariance (ANCOVA). A number of controls were expected to suffer from EDs, therefore all participants who achieved an EDI-2 Symptom Index above the validated cut-off value of 21 [32] were considered in the patient group in statistical analyses. This was done to better describe the sample and affected only comparisons between groups as SEM analysis was performed on the whole sample. A total of 42 control participants were considered in the ED group.

The structural equation modelling (SEM) technique was used in order to test the proposed model and pathways between variables. In particular, the hypothesized model featured a serial mediation with four steps, in which the PIU (IUQ, step 1) increased the domain relating to over-investment in the physical aspect (step 2), with the components of appearance-related importance (ASI-R) and comparisons (SACS); the latter led to higher levels of body uneasiness (BUT-A, step 3), which, in turn, maintained ED symptoms (step 4). This last step was modelled as two latent variables: one related to interpersonal difficulties (ID, with loadings on EDI-2 Social Insecurity and Interpersonal Distrust) and the other to ED-specific psychopathology (EDP, with loadings on EDI-2 Drive for Thinness, Bulimia, Body Dissatisfaction and Interoceptive Awareness). For EDP, in addition to the three main EDI-2 subscales (components of the EDI-2 Symptom Index) Interoceptive Awareness was also included, as a dimension considered central in EDs with excellent sensitivity and specificity properties [53, 54].

The variables of each step were regressed on all those of the previous steps, in accordance with the theory of serial mediation. In addition, given the inverse association between age and social media use [55], the model was adjusted for these potentially confounding effects by entering age as a covariate in all equations. The following regression equations were used for the final model:

$$\begin{aligned} & {\text{IUQ }} = {\text{ intercept }} + {\text{ b}}_{{{\text{Age}} - {\text{IUQ}}}} *{\text{Age}} \\ & {\text{SACS }} = {\text{ intercept }} + {\text{ b}}_{{{\text{IUQ}} - {\text{SACS}}}} *{\text{IUQ }} + {\text{ b}}_{{{\text{Age}} - {\text{SACS}}}} *{\text{Age}} \\ & {\text{ASI}} - {\text{R }} = {\text{ intercept }} + {\text{ b}}_{{{\text{IUQ}} - {\text{ASI}}}} *{\text{IUQ }} + {\text{ b}}_{{{\text{Age}} - {\text{ASI}}}} *{\text{Age}} \\ & {\text{BUT }} = {\text{ intercept }} + {\text{ b}}_{{{\text{IUQ}} - {\text{BUT}}}} *{\text{IUQ }} + {\text{ b}}_{{{\text{SACS}} - {\text{BUT}}}} *{\text{SACS }} + {\text{ b}}_{{{\text{ASI}} - {\text{BUT}}}} *{\text{ASI }} + {\text{ b}}_{{{\text{Age}} - {\text{BUT}}}} *{\text{Age}} \\ & {\text{ID }} = {\text{ b}}_{{{\text{IUQ}} - {\text{ID}}}} *{\text{IUQ }} + {\text{ b}}_{{{\text{SACS}} - {\text{ID}}}} *{\text{SACS }} + {\text{ b}}_{{{\text{ASI}} - {\text{ID}}}} *{\text{ASI }} + {\text{ b}}_{{{\text{BUT}} - {\text{ID}}}} *{\text{BUT }} + {\text{ b}}_{{{\text{Age}} - {\text{ID}}}} *{\text{Age}} \\ & {\text{EDP }} = {\text{ b}}_{{{\text{IUQ}} - {\text{EDP}}}} *{\text{IUQ }} + {\text{ b}}_{{{\text{SACS}} - {\text{EDP}}}} *{\text{SACS }} + {\text{ b}}_{{{\text{ASI}} - {\text{EDP}}}} *{\text{ASI }} + {\text{ b}}_{{{\text{BUT}} - {\text{EDP}}}} *{\text{BUT }} + {\text{ b}}_{{{\text{Age}} - {\text{EDP}}}} *{\text{Age}} \\ \end{aligned}$$

IUQ, SACS and EDI-2 scores were divided by 10, in order to facilitate model convergence; results and regression coefficients should be interpreted accordingly. Moreover, the following variables were allowed to covary in the model: SACS with ASI-R; ID with EDP; EDI-2 Bulimia with Interoceptive Awareness and Drive for Thinness; EDI-2 Body Dissatisfaction and Interoceptive Awareness with both ID manifest variables. The scaling of latent variables was defined using the marker-variable method. Standardized estimates of all parameters were computed for both observed and latent variables (completely standardized solution). SEM analysis was performed using the maximum-likelihood estimator with robust Huber-White standard errors, scaled test statistic and robust fit measures (MLR estimator). This method produces errors, test statistics and fit measures that are robust to non-normality, and can manage incomplete data. Model fit was assessed by computing the following commonly used fit measures: Comparative Fit Index (CFI ≥ 0.95 for good fit), Tucker–Lewis Index (TLI ≥ 0.95 for good fit), Root Mean Square Error of Approximation (RMSEA ≤ 0.06 for good fit), Standardized Root Mean Square Residual (SRMR ≤ 0.08 for good fit) [56]. Finally, all possible indirect effects of PIU on ED symptoms were tested by computing bias-corrected bootstrapped confidence interval (CI), with 10,000 resamples; the mediation effect was considered statistically significant if the CI did not include zero.

The a priori theoretic model did not predict clear-cut differences between ED patients and controls regarding the relationships between variables; instead, all subjects were expected to be arranged along a continuum extending from minor to major levels of psychopathology. Consequentially, the SEM analysis was performed on the whole sample. Nested model comparison was used to test for any differences between ED patients and controls in terms of the effects of interest (factor loadings and regression coefficients): a non-statistically significant scaled Chi-Squared difference test indicates the invariance of the coefficients across groups and supports using the whole sample for the main analysis. A similar approach using nested model comparison was used to test the role of age as a meaningful covariate: a significant Chi-squared difference test indicates that an age-adjusted model performs better than a simpler unadjusted one. Finally, an alternative model was tested in which the ASI-R and IUQ scores were swapped with respect to the original model (and therefore ASI-R predicted IUQ and SACS): the two models were compared using Vuong’s comparison test for non-nested models.

All analyses were performed using R statistical software version 4.1.2 [57] and the following libraries: dplyr [58], lavaan [59].

## Results

The final sample consisted of 152 individuals affected by an ED, and 234 control subjects. General and psychopathological characteristics of the sample are reported in Table 1. Patients suffering from EDs showed higher levels of psychopathology and body uneasiness than controls and scored higher in SACS and ASI-R questionnaires, indicating greater individual psychological investment in physical appearance and propensity for appearance comparison (Table 1). Moreover, patients also reported more PIU with respect to controls, as evidenced by higher IUQ scores (Table 1).

**Table 1:** Characteristics of the sample, divided by group

	Controls (*n* = 234)	Patients with ED (*n* = 152)	F
Age (years)	25.66 ± 5.45	26.70 ± 8.34	2.19
Education (years)	15.21 ± 1.72	14.67 ± 2.26	7.53**
BMI (kg/m^2^)	21.24 ± 3.01	22.69 ± 6.40	6.63*
Daily Instagram use (min)	97.20 ± 62.89	119.07 ± 75.17	8.48**
IUQ total score	16.89 ± 5.71	18.75 ± 7.41	9.71**
SACS total score	10.57 ± 4.86	13.99 ± 4.80	41.85***
ASI-R total score	3.12 ± 0.61	3.82 ± 0.65	121.03***
BUT-A			
Weight phobia	1.08 ± 0.86	3.32 ± 0.98	557.14***
Body image concerns	0.95 ± 0.78	2.99 ± 1.16	420.55***
Avoidance	0.35 ± 0.54	1.99 ± 1.29	288.32***
Compulsive self-monitoring	0.72 ± 0.70	2.22 ± 1.32	223.98***
Depersonalization	0.42 ± 0.60	2.08 ± 1.38	258.62***
Global severity index	0.75 ± 0.61	2.61 ± 1.02	498.30***
EDI-2			
Ineffectiveness	4.03 ± 4.54	12.86 ± 7.56	201.16***
Maturity fears	5.26 ± 4.33	8.52 ± 5.87	38.67***
Social insecurity	5.39 ± 3.55	8.06 ± 4.08	44.63***
Body dissatisfaction	6.17 ± 4.62	17.50 ± 5.99	433.87***
Perfectionism	3.04 ± 2.97	6.02 ± 4.10	69.45***
Interpersonal distrust	3.79 ± 3.58	7.31 ± 4.51	71.40***
Impulse regulation	1.94 ± 3.46	7.88 ± 6.68	130.35***
Drive for thinness	1.88 ± 2.82	14.67 ± 4.90	1046.23***
Bulimia	0.53 ± 1.15	6.86 ± 5.96	247.36***
Interoceptive awareness	2.37 ± 3.06	11.71 ± 7.18	312.17***
Asceticism	2.94 ± 2.06	7.76 ± 4.52	204.20***

Results for age-adjusted comparisons between groups are reported

*ASI-R* Appearance Schema Inventory-Revised, *BMI* body mass index, *BUT-A* Body Uneasiness Test A, *ED* eating disorder, *EDI-2* Eating Disorder Inventory 2, *IUQ* Instagram Use Questionnaire, *SACS* State Appearance Comparison Scale

^*^p < 0.05, **p < 0.01, ***p < 0.001

### SEM analysis

**Fig. 2:**
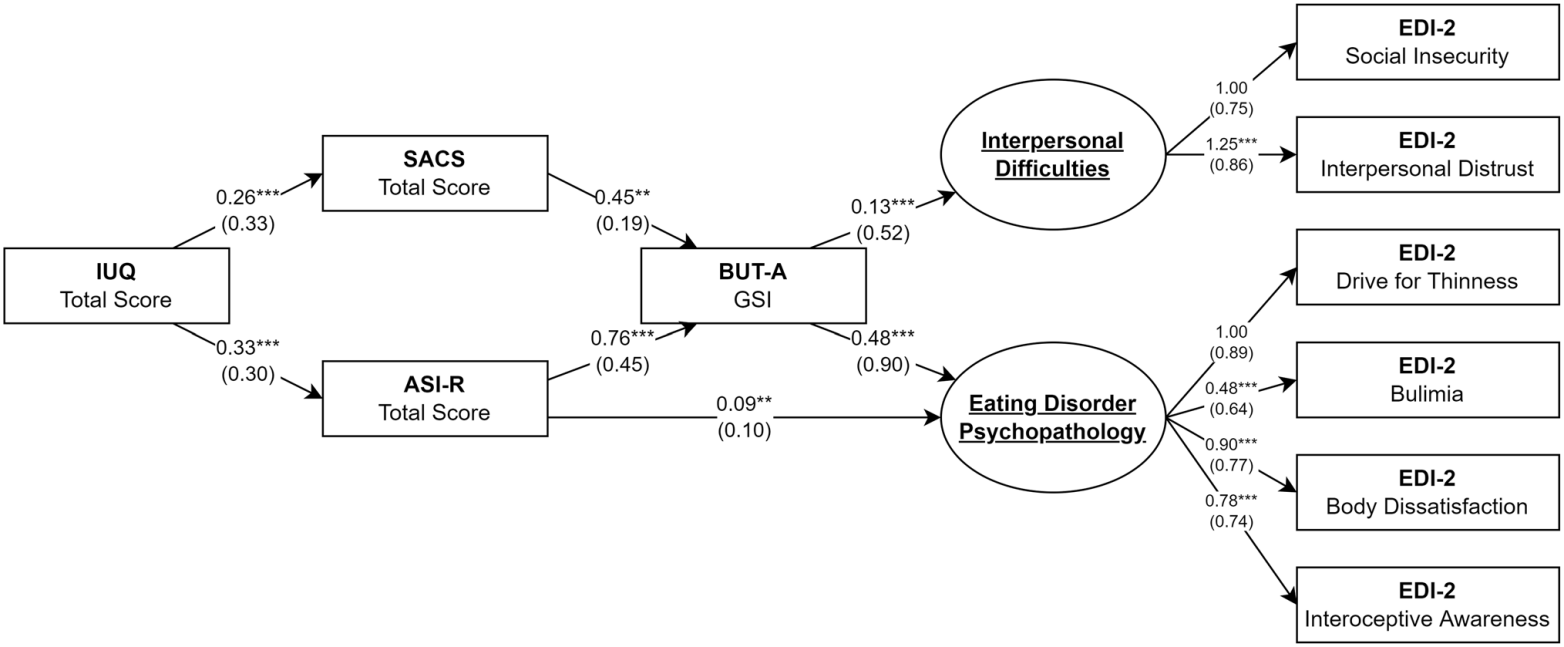
Results of the SEM analysis. Rectangles represent observed variables; circles represent latent variables. Regression effects and loadings are illustrated as single-headed arrows, together with their respective unstandardized coefficients; standardized coefficients are reported in parenthesis. **p < 0.01, ***p < 0.001. *ASI-R* Appearance Schema Inventory-Revised, *BUT-A GSI* Body Uneasiness Test A Global Severity Index, *EDI-2* Eating Disorder Inventory 2, *IUQ* Instagram Use Questionnaire, *SACS* State Appearance Comparison Scale

Results are shown in Fig. 2. The computed robust fit measures confirmed the good fit (CFI = 0.987, TLI = 0.968, RMSEA = 0.054, SRMR = 0.027), confirming that the collected data were consistent with the hypothesized model. PIU significantly predicted greater psychological investment in physical appearance and propensity for appearance comparison, which in turn correlated with higher levels of body uneasiness (Fig. 2). Both ED-related latent factors were strongly associated with body uneasiness, with ED-specific psychopathology also predicted by ASI-R score (Fig. 2). Age was a significant covariate in the equations predicting PIU (b = − 0.03, p < 0.001) and interpersonal difficulties (b = 0.01, p = 0.048), and did not show an association with SACS (b < 0.01, p = 0.936), ASIR (b = 0.01, p = 0.335), BUT (b = 0.02, p = 0.081) or ED psychopathology (b = 0.01, p = 0.135). A simpler model that was not adjusted for age performed significantly worse (Δχ^2^ = 11.80, p = 0.038).

To confirm the appropriateness of using the whole sample for the main analysis, the invariance of factor loadings and regression coefficients across groups (patients with ED and controls) was tested using nested model comparison. The scaled Chi-Squared difference test was not statistically significant (Δχ^2^ = 25.85, p = 0.308), indicating that a more complex model where loadings and coefficients were estimated separately for each group did not lead to an appreciable improvement with respect to a simpler model with equal effects.

Mediation analysis confirmed the presence of an indirect effect of PIU on ED symptoms, through multiple mediation pathways. Most of the indirect effect was from the increase in levels of body uneasiness associated with the greater propensity for appearance comparison (b_IUQ-SACS_*b_SACS-BUT_*b_BUT-EDP_ = 0.06, 95% CI [0.01, 0.11]) and psychological investment in physical appearance (b_IUQ-ASI_*b_ASI-BUT_*b_BUT-EDP_ = 0.12, 95% CI [0.07, 0.19]). However, part of the indirect effect was independent of BUT scores and was mediated only by higher ASI-R scores (b_IUQ-ASI_*b_ASI-EDP_ = 0.03, 95% CI [0.01, 0.06]). The serial mediation pathways involving higher levels of body uneasiness were similar for interpersonal difficulties (b_IUQ-SACS_*b_SACS-BUT_*b_BUT-ID_ = 0.02, 95% CI [0.01, 0.03]; b_IUQ-ASI_*b_ASI-BUT_*b_BUT-ID_ = 0.03, 95% CI [0.02, 0.06]). However, no indirect effects independent of BUT scores were found. The overall coefficients of determination were R^2^ = 0.89 for EDP and R^2^ = 0.30 for ID.

The proposed model was finally compared with an alternative model in which the ASI-R and IUQ scores were swapped, in order to further confirm its validity. The alternative model had worse fit indices (CFI = 0.971, TLI = 0.929, RMSEA = 0.082, SRMR = 0.038) with a higher Bayesian information criterion (BIC_Original Model_ = 8055.83, BIC_Alternative Model_ = 8072.68), and Vuong’s tests confirmed that it performed significantly worse than the proposed one (z = 2.07, p = 0.019).

## Discussion

To the best of our knowledge, this is the first study that evaluated the interplay between Problematic Instagram Use (PIU) and EDs psychopathology by focusing on potential explanatory mechanisms. As expected, according to the first hypothesis, women with ED symptoms showed higher levels of PIU as well as greater propensity for appearance comparison engendered by exposure to Instagram images with respect to women without ED symptoms. In addition, this finding is accompanied by a reported increased time spent on Instagram by women suffering from EDs. This result confirms the previously reported association between the intensity of Instagram use and disordered eating [31], and extends previous findings evidencing for the first time the relevance of problematic Instagram use in EDs.

The second aim of the current study was to evaluate a model explaining how PIU relates to EDs psychopathology on young women, considering the mediating role of specific dimensions of body image (i.e., psychological investment in physical appearance and body uneasiness) and appearance comparison tendency. Testing the hypothesis within the SEM framework allowed the simultaneous study of multivariate regression pathways with robust statistical methods, confirming the goodness of fit of the a priori hypothesized model (illustrated in Fig. 1) with the collected data. Moreover, the use of latent variables for the investigation of constructs related to ED-specific psychopathology represents a major strength of the study. Indeed, PIU predicts greater psychological investment in physical appearance and more frequent appearance comparisons, which in turn lead to body uneasiness. Furthermore, body uneasiness is associated with both latent constructs related to ED-specific psychopathology: ED symptoms and related interpersonal difficulties. The relationship between generalized problematic SNS use and EDs psychopathology has been previously reported [60]. The current study extends these previous findings by focusing attention on the problematic use of a specific image-centered SNS like Instagram, on the one hand, and considering the mediating role of both cognitive and affective dimensions of body image, alongside with appearance comparisons made to Instagram images, on the other.

Indeed, PIU is associated with ED symptoms and interpersonal-related difficulties indirectly through two mediational pathways: (i) the multiple/serial mediation effect of appearance comparison and body uneasiness (ii) the multiple/serial mediation effect of psychological investment in physical appearance and body uneasiness. Moreover, for ED symptoms an additional simple mediation effect of psychological investment in physical appearance independent of body uneasiness was found.

On the one hand, the present study supports previous findings on the mediating role of appearance comparison in the relationship between exposure to Instagram images and body image concern [32]. Indeed, the association between PIU and body uneasiness is mediated by appearance comparison, which in turn predicts ED symptoms and related interpersonal difficulties. On the other hand, our findings found first evidence for the role of psychological investment in physical appearance as mediator of the relationship between PIU and body uneasiness, which in turn leads to ED symptoms and interpersonal difficulties. Moreover, the psychological importance one places on one’s appearance enhanced by PIU predicts ED symptoms also independently of body uneasiness. This result highlights the importance of considering cognitive dimensions of body image alongside with affective and behavioral ones when investigating the effect of excessive use of Instagram on psychological outcomes. In fact, the compulsive use of Instagram could prompt to disordered eating outcomes as a consequence of a continuous exposure to idealized beauty images. This may lead women to compare themselves to these attainable images, with the result of attaching more psychological importance to their physical appearance (i.e., using appearance as a self-defining feature and managing one’s own appearance for esthetic purposes), and feeling worried about their body shape and weight. Interestingly, all these mechanisms could lead not only to ED-specific psychopathological domains (i.e., drive for thinness, bulimia, body dissatisfaction and interoceptive awareness) but also to interpersonal difficulties (i.e., social insecurity and interpersonal distrust). These findings confirmed previous preliminary evidence reporting that excessive Instagram use may be related to higher self-reported loneliness, fear of missing out, and lack of social support [31].

### Limitations and future research

The present study has some limitations that need to be considered. First, the data was solely based on self-report questionnaires, which may be subject to social desirability and self-report biases. Second, the Italian versions of the Instagram Use Questionnaire, and the State Appearance Comparison Scale, were used, and although all the scales demonstrated good psychometric properties in previous preliminary studies conducted in Italy, further testing is necessary to ensure the reliability of these instruments. Third, the convenience sampling technique and the recruitment of a sample consisting only of females prevent the generalizability of the results to the entire population. Finally, given the correlational nature of the study design, it was not possible to draw causal inferences. Longitudinal studies are needed to clarify the directions of the correlations that were found in our study. It needs to be established whether problematic Instagram use causes ED symptoms, or else whether excessive Instagram use is an indicator of existing psychological problems.

### Implications

Notwithstanding the above limitations, the current study extends our understanding of the role of PIU in EDs development and maintenance and has potential clinical implications. The study provides empirical evidence that, to prevent and/or reduce young women ED psychopathology, health professionals and clinicians should also focus on patients’ Instagram use and related body image concerns. In terms of assessment of patients with EDs, information should be collected in relation to (excessive) Instagram use, and their propensity to subsequently compare their own appearance to the appearance of the bodies depicted in this content, and to attach more importance to their own appearance as a consequence of excessive Instagram use, as they could represent maintaining factors of body image concerns and ED psychopathology. The current findings could also be used by clinicians to provide patients with psychoeducation about the potential negative effects of maladaptive Instagram use. Social media literacy programs aimed at reducing social comparisons with unrealistic images that idealize thinness, fitness and appearance perfection, and its negative effect on body image dimensions among young women, might reduce the effect of PIU in maintaining EDs symptoms.

## Conclusion

In conclusion, the present study provides the first evidence of the relationship between PIU and ED psychopathology among a sample of young women. Moreover, an explanatory model of how ED symptoms could be drawn and maintained by an addictive use of Instagram was proposed. Appearance comparison tendency, psychological investment in physical appearance, and body uneasiness play an important mediating role.

## Data Availability

The datasets generated during and/or analyzed during the current study are available from the corresponding author on reasonable request.
